# Compression of left renal vein and left common iliac vein on CT scans: how often are they detected?

**DOI:** 10.1590/1677-5449.190121

**Published:** 2020-06-01

**Authors:** Adenauer Marinho de Oliveira Góes, Rafaela de Souza Araújo, Ismari Perini Furlaneto, Waldonio de Brito Vieira

**Affiliations:** 1 Centro Universitário do Estado do Pará – CESUPA, Habilidades Cirúrgicas e Cirurgia Vascular, Belém, PA, Brasil.; 2 Centro Universitário do Estado do Pará – CESUPA, Curso de Medicina, Belém, PA, Brasil.; 3 Clínica DIA/Hospital Amazônia, Radiologia, Belém, PA, Brasil.

**Keywords:** nutcracker syndrome, May-Thurner syndrome, computed tomography, iliac vein, compression

## Abstract

**Background:**

The nutcracker and May-Thurner syndromes are rare and, although often underdiagnosed, they can cause limiting symptoms. They are frequently considered only after exclusion of other diagnoses and there is no consensus in the literature on prevalence, incidence, or diagnostic criteria.

**Objectives:**

To estimate the frequency of compression of the left common iliac vein and left renal vein in CT scans of the abdomen and pelvis.

**Methods:**

Descriptive, quantitative, cross-sectional study. The criteria used to define compression of the left renal vein were a hilar/aortomesenteric diameter ratio > 4 and aortomesenteric angle < 39° and the criterion for compression of the left common iliac vein was a diameter < 4mm.

**Results:**

CT scans of 95 patients were analyzed; 61% were women and 39% were men. Left renal vein compression was observed in 24.2% of the sample, with a mean age of 48.8 years, occurring in 27.6% of the women and 18.9% of the men (p = 0.3366). Compression of the left common iliac vein was detected in 15.7% of the sample, with a mean age of 45.9 years, occurring in 24.1% of the women and 2.7% of the men (p = 0.0024). Both veins were compressed in 7.4% of the patients.

**Conclusions:**

Left renal vein compression was detected in women and men at similar frequencies, whereas left common iliac vein compression was more frequent in women. Both venous compressions were most frequently found in patients aged 41 to 50 years.

## INTRODUCTION

The nutcracker syndrome is considered rare. It consists of a set of signs and symptoms caused by compression of the left renal vein (LRV) because of an acute angle between the abdominal aorta and the superior mesenteric artery.^1^^,^^2^ A less common variant is caused by a retroaortic LRV, compressed between the aorta and a vertebral body (posterior nutcracker syndrome).^3^^,^^4^ The syndrome was first described by Schepper^5^ in 1972 and its most common clinical findings are hematuria, pelvic pain, pelvic varicose veins, and orthostatic proteinuria.^6^^,^^7^ It can course with chronic pelvic pain, infertility, and renal failure.^8^^,^^9^ Venous compression detected radiologically, but not associated with symptoms, is called the nutcracker phenomenon.

Computed tomography (CT) with intravenous contrast is often used for radiological diagnosis, because it is a noninvasive examination, relatively inexpensive, and widely available. On CT, the aortomesenteric angle can be measured in the sagittal plane and the ratio between the hilar and aortomesenteric diameters of the LRV can be determined in the axial plane.^10^^,^^11^ An aortomesenteric angle smaller than 39°^12^^,^^13^ and a ratio between the hilar/aortomesenteric diameters exceeding 4 are considered diagnostic criteria for compression of the LRV.^6^^,^^14^

Another anatomic situation that can cause vein compression in compression of the left common iliac vein (LCIV) by the right common iliac artery against the vertebral body, which was first described in 1857 by Virchow^15^ and later defined as a “syndrome” and described in detail in a study published by May and Thurner^16^ in 1958. The main clinical findings are iliofemoral deep venous thrombosis, pain, varicose veins, edema, venous eczema, and venous stasis ulcers involving the left lower limb.^8^^-^10 CT has high sensitivity and specificity for diagnosis of this syndrome.^8^

Classically, May-Thurner Syndrome is described as more prevalent among women in their third or fourth decades of life and could be associated with up to 49% of cases of deep venous thrombosis (DVT) in the left lower limb.^4^^,^^15^^,^^17^ In the pioneering studies by May and Thurner,^16^ a 22% prevalence of venous compression was found in the 430 cadavers analyzed.

The prevalence of nutcracker syndrome remains debatable because of the lack of uniform diagnostic criteria and the wide variety of symptoms. Some studies have reported equal prevalence in both sexes and a predominance of occurrence among young people with low body mass index.^10^^,^^12^

The objectives of this study were to estimate the frequency of compression of the left renal and common iliac veins in patients who underwent CT of abdomen and pelvis; to determine whether detection of compression of these veins is more frequent in a given sex or age group; to evaluate the diameters of the respective veins in patients with and without the criteria for compression; and to determine which of the radiological criteria for compression of the LRV is found more frequently.

## METHODS

A quantitative, descriptive, cross-sectional study was conducted to determine the prevalence of compression of the LRV (nutcracker phenomenon) and compression of the LCIV in CT scans of the abdomen and pelvis conducted between January 2017 and January 2018. The sample was selected by convenience from all examinations made available by a radiology service affiliated to a teaching institution that corresponded to the period studied, after application of inclusion and exclusion criteria. The CT scans were conducted with intravenous contrast on a 16-channel GE Healthcare scanner with a 512 × 512 resolution matrix and slice thickness of 1.25 mm.

The inclusion criteria were CT scans performed with intravenous contrast on patients of either sex with a minimum age of 18 years. Exclusion criteria included tomographic findings suggestive of malignancy that could contribute to venous compression, renal or pelvic venous malformations, and presence of stents in the LCIV or LRV.

With the aid of RadiAnt DICOM viewer 4.6.9 software, the ratio between the diameter of the LRV at the hilar level and at the level of the aortomesenteric angle was calculated on axial slices and the aortomesenteric angle was measured on sagittal slices. For the LCIV, the smallest smaller diameter between the right common iliac artery and the adjacent vertebral body was measured.

The criteria adopted to define compression of the LRV as present were a hilar/aortomesenteric diameter ratio exceeding 4 and an aortomesenteric angle smaller than 39°. The criterion considered for compression of the LCIV was a diameter of less than 4 mm.

Normality of distributions was verified using the D’Agostino-Pearson test. Student’s *t* test for independent samples was used for parametric distributions. The Mann-Whitney test, the chi-square test of adherence, or the G test for independent samples were used for nonparametric distributions. All tests were run using BioEstat 5.4, and a p value of ≤ 0.05 was adopted as the criterion for statistical significance. The study was approved by the institutional ethics committee under protocol number 2.683.725.

## RESULTS

A total of 95 CT scans of the abdomen and pelvis were analyzed, although two patients were excluded from the analysis of aortomesenteric angle because they had a retroaortic LRV. The mean of age of patients was 53.70 years ± 14.90 years, ranging from 21 to 83 years. The most prevalent age group was 61 years or over (p = 0.0002; Table 1). Although female patients predominated in the sample as a whole (p = 0.0312; Table 1), there were no statistically significant differences between the proportions of men and women in each age group studied (p = 0.5295; data not shown).

**Table 1:** Distribution of patients by sex and age group.

**Variable**	**n**	**%**	***p*-value** ^*^
Sex			
Male	37	39.00	0.0312†
Female	58	61.00	
Age group (years)			
21-30	08	8.40	0.0002^†^
31-40	11	11.60	
41-50	18	18.90	
51-60	25	26.30	
≥ 61	33	34.80	

* Chi-square test of adherence;

† Statistically significant;

n: number of patients.

Table 2 lists the mean diameters of the LRV at the level of the hilar and the aortomesenteric angle, the mean value for the hilar/aortomesenteric diameter ratio, the mean aortomesenteric angle, and the mean LCIV diameter at the point of greatest compression. With regard to compression of the LRV, an aortomesenteric angle < 39° was observed in 22 of 93 patients (23.70%) and a hilar/aortomesenteric diameter ratio > 4 was observed in 2 of 95 (2.10%) patients. One of 95 (1.10%) patients was positive for both criteria, making a total of 23 out of 95 (24.2%) patients with one of more criteria that define the nutcracker phenomenon. Compression of the LCIV (diameter < 4 mm) was identified in 15 of 95 (15.80%) patients. In 7 out of 95 (7.4%) patients, one or more tomographic criteria were detected for compression of both the LRV and the LCIV.

**Table 2:** Diameters of the left renal and left common iliac veins, ratio between the diameters of the left renal vein at the hilar segment and at the level of the aortomesenteric angle, and aortomesenteric angle values.

**Variable**	**Mean ± standard deviation**	**Minimum-maximum**
Hilar LRV diameter (mm)	8.37±1.94	3.25-13.40
Aortomesenteric LRV diameter (mm)	6.63±2.58	1.18-16.10
Hilar/aortomesenteric diameter ratio	1.53±0.93	0.51-6.66
Aortomesenteric angle (degrees)	61.12±24.53	17.60-124.70
Diameter LCIV (mm)	7.74±3.89	1.31-22.80

mm = millimeters; LRV = left renal vein; LCIV = left common iliac vein.

Table 3 shows a comparison of the hilar/aortomesenteric diameter ratio and the aortomesenteric angle in patients with and without compression of the LRV. It is notable that the hilar/aortomesenteric diameter ratio was significantly smaller among patients with compression of the LRV (p < 0.0001) and the aortomesenteric angle was significantly larger among patients without LRV compression (p < 0.0001).

**Table 3:** Comparisons of hilar/aortomesenteric diameter ratio and aortomesenteric angle in patients with and without compression of the left renal vein.

**Variable**	**Compression of the left renal vein**		***p*-value**
**Present**	**Absent**
**Hilar/aortomesenteric diameter ratio**	**n = 23**	**n = 72**	
Mean ± standard deviation	2.53±1.29	1.21±0.42	< 0.0001^†^
Minimum-maximum	0.69-6.66	0.51-2.51	
95%CI	1.97-3.09	1.11-1.30	< 0.0001†
**Aortomesenteric angle (degrees)**	**n = 23**	**n = 70** a	
Mean ± standard deviation	32.71±15.43	70.45±19.21	
Minimum-maximum	17.60-97.70	41.00-83.70	
95%CI	26.03-39.38	65.87-75.03	

Mann-Whitney test.

† Statistically significant;

n: number of patients;

a n = 2 patients excluded from this comparison because they had a retroaortic left renal vein; 95%CI = 95% confidence interval.

With regard to the relationship between presence of LRV compression and sex, no statistically significant difference was observed (p = 0.3666; Table 4). Mean age of patients with and without compression was similar (p = 0.0666; Table 5).

**Table 4:** Distribution of patients by sex and presence of venous compressions investigated.

**Variable**	**Sex**		***p*-value**
	**Female n; %**	**Male n; %**	
**LRV compression**			
Present	16; 27.60	07;18.90	0.3366
Absent	42; 72.40	30; 81.10	
**Compression of LCIV**			
Present	14; 24.10	01; 2.70	0.0024†
Absent	44; 75.90	36; 97.30	
**Both compressions**			
Present	06; 10.30	01; 2.70	-

G test of independence. n: number of patients.

† Statistically significant; LRV = left renal vein; LCIV = left common iliac vein.

**Table 5:** Mean, minimum, and maximum values and standard deviation of age by presence of the compressions investigated.

**Variable**	**Age (years)**	**Minimum-maximum**	
	**Mean ± standard deviation**		***p*-value**
**LRV compression**			
Present	48.80±17.90	21-83	0.0666
Absent	55.30±13.50	24-82	
**Compression of the LCIV**			
Present	45.90±15.20	24-77	0.0248†
Absent	55.20±14.40	21-83	
**Both compressions**			
Present	51.60±16.50	26-77	0.6924
Absent	53.90±14.80	21-83	

Student’s *t* test.

† Statistically significant; LRV = left renal vein; LCIV = left common iliac vein.

Table 6 shows that the diameter of the LCIV was significantly smaller among patients classified as having compression of this vein (p < 0.0001). Compression of the LCIV was detected with a significantly higher frequency in women (p = 0.0024), and the mean of age of patients who exhibited this phenomenon was significantly lower than the mean age of those who did not (p = 0.0248; Tables 4 and 5).

**Table 6:** Comparison of diameter of the left common iliac vein in patients with and without compression of the left common iliac vein.

**Variable**	**Compression of the left common iliac vein**		***p*-value** *
	**Present**	**Absent**	
**Diameter of the left common iliac vein (mm)**	n = 15	n = 80	
Mean ± standard deviation	2.69±0.76	8.69±3.48	< 0,0001†
Minimum-maximum	1.31-3.90	4.43-22.80	
95%CI	2.26-3.10	7.92-9.46	

* Mann-Whitney test;

n: number of patients;

† Statistically significant; 95%CI = 95% confidence interval; mm = millimeters.

## DISCUSSION

Although radiological identification of these compressive phenomena was analyzed in this study, it was not possible to establish any clinical correlations, because only access to CT images was available. Of a total of 95 CT scans analyzed, 58 were of women (61%) and 37 of men (39%). This proportion between sexes is similar to that reported in several studies of CT scans covering the same subject, such as one by Zhong et al.,^18^ who studied a sample comprising 75% women and 25% men, and another by Narayan et al.,^19^ whose sample composition was 59% women and 41% men. According to Levorato et al.,^20^ this pattern may be because women seek health services more often than men.

Reviewing the literature on nutcracker syndrome, it was observed that there is a considerable variation in the cutoff points adopted for the aortomesenteric angle, with studies that adopted angles ranging from 25 to 45°,^4^^,^^7^^,^^8^^,^^12^^,^^13^^,^^21^^,^^22^ while hilar/aortomesenteric diameter ratios were used varying from > 4 to > 4.9.^6^^,^^7^^,^^12^^-^14^,^^21^ The criteria for LRV compression adopted in the present study were a hilar/aortomesenteric diameter ratio of > 4 and an aortomesenteric angle of < 39°.

Some studies describe LRV compression as more frequent among young women in their second to fourth decades of life.^3^^,^^4^^,^^8^^,^^9^^,^^23^ However, other studies suggest that there is no statistically significant difference in sex distribution, either for the nutcracker syndrome or for the nutcracker phenomenon.^11^^,^^12^^,^^18^^,^^21^^,^^22^^,^^24^^,^^25^ Our study did not demonstrate a statistically significant difference in occurrence between the sexes. Yun et al.^21^ demonstrated that venous compression was present in 37.5% of a sample of patients; while prevalence was 10.4% in a study by Poyraz et al.^11^ This difference may be because of the lack of uniformity in the cutoff points used by different authors. In our study, a frequency of 24.2% was observed.

In the study by Yun et al.,^21^ the mean age of patients with LRV compression was 36.8 ± 14.3 years, and in a study by Kim et al.,^13^ mean age was 23.9 ± 4.6 years. The mean age we found for patients with LRV compression was 48.8 years.

The mean aortomesenteric angle observed in the study by Yun et al.^21^ was 20° in patients with the nutcracker syndrome and 25° in asymptomatic patients, while Zhong et al.^18^ detected an angle of 32.3° ± 7.6° in patients with nutcracker syndrome. In our study, the mean aortomesenteric angle among patients with compression was 27.3°, which is similar to results that can be found in the literature.

We only observed a hilar/aortomesenteric diameter ratio greater than 4 in 2 patients, whereas an aortomesenteric angle smaller than 39° was detected in 22 of the 93 patients for whom this analysis was possible, suggesting that this criterion has greater sensitivity. Since the hilar/aortomesenteric diameter ratio criterion is found with lower frequency, it may be more specific and, as a result, may be attributed greater value when detected in patients with clinical presentation compatible with the syndrome. This large difference in the frequencies of the two criteria may be because we standardized on an aortomesenteric angle < 39° for LRV compression. If we had adopted smaller angles, as other authors have done, this disparity may have been smaller. However, the values adopted diverge widely in the literature^4^^,^^7^^,^^8^^,^^12^^,^^13^^,^^21^^,^^22^ and this is one of the points on which there is still no consensus with relation to this subject.

Zhong et al.^18^ observed a mean hilar/aortomesenteric diameter ratio of 3.4, while Kim et al.^13^ reported that the mean hilar/aortomesenteric diameter ratio in their study was 5 ± 1.7 (both studies were of symptomatic patients with the nutcracker syndrome). In our study, the mean hilar/aortomesenteric diameter ratio among patients considered as having compression (because they had an aortomesenteric angle < 39°) was 2.1.

This disagreement can be attributed to the fact that the patients in the studies cited above had diagnoses of nutcracker syndrome, whereas the patients in our study were assessed on the basis of incidental radiological findings of compression. No studies were found that compare the hilar/aortomesenteric diameter ratio between individuals with and without symptoms.

**Figure 1:**
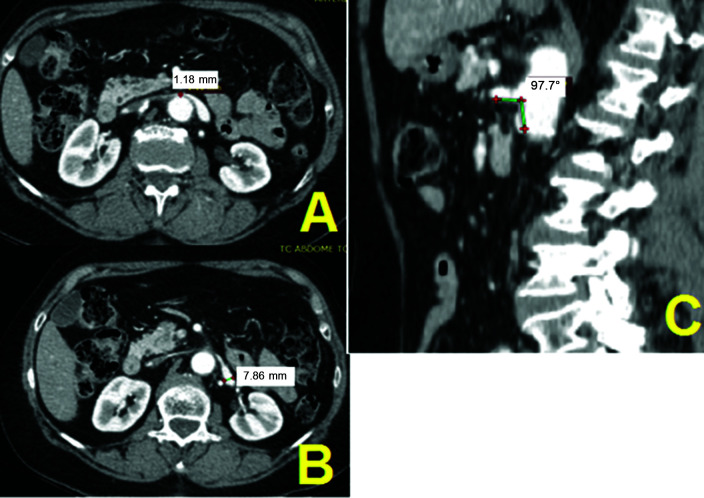
Computed tomography with intravenous contrast (patient n 67). (A) Diameter of the left renal vein at the renal hilum; (B) Diameter of the left renal vein at the level of the aortomesenteric angle; (C) Measurement of the aortomesenteric angle on a sagittal slice.

In one patient, we detected LRV compression without narrowing of the aortomesenteric angle, as is classically reported (the patient whose CT images are shown in Figure 1). This finding may be because of duodenal interposition, described as a cause of LRV compression with a normal aortomesenteric angle.^14^

**Figure 2:**
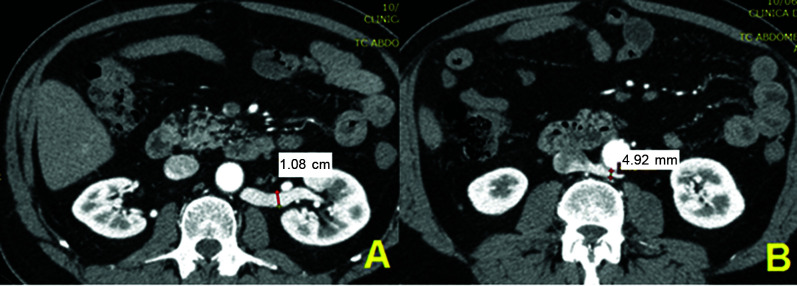
Computed tomography with intravenous contrast (patient n 54). (A) Diameter of the left renal vein at the renal hilum; (B) Diameter of the left renal vein at the retroaortic position.

In two patients, an anatomic variant with retroaortic LRV was detected, as shown in Figure 2. This variation means that compression can only be evaluated by calculating the ratio between the hilar diameter and the diameter of the vein at the point of maximum compression between the aorta and the adjacent vertebra, since the LRV of these patients does not follow a path through the aortomesenteric angle.^6^ These patients were excluded from the calculations involving the aortomesenteric angle, but, because the hemodynamic mechanism is similar, they were evaluated according to the same cutoff point for hilar/aortomesenteric diameter ratio used for the remainder of the patients.

**Figure 3:**
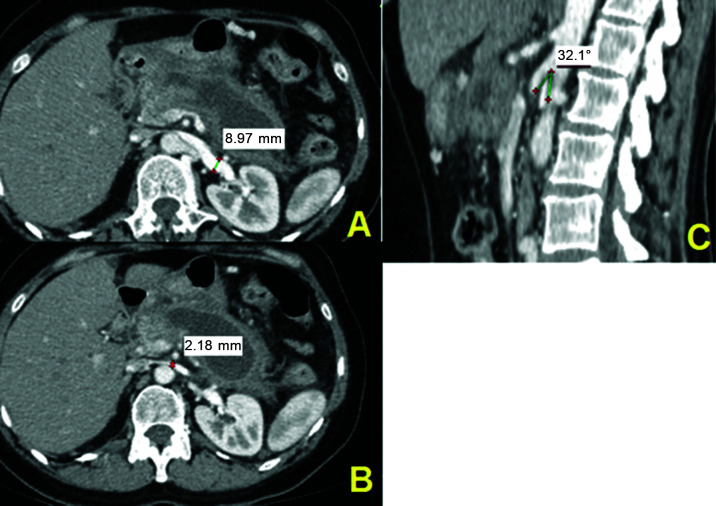
Computed tomography with intravenous contrast (patient n 71). (A) Diameter of the left renal vein at the renal hilum; (B) Diameter of the left renal vein at the level of the aortomesenteric angle; (C) Measurement of the aortomesenteric angle on a sagittal slice.

We only detected a hilar/aortomesenteric diameter ratio of less than 4 in two patients. Just one patient was positive according to both criteria, shown in Figure 3.

The May-Thurner Syndrome is caused by compression of the LCIV between the right common iliac artery and the adjacent lumbar vertebra,^26^^-^28 provoking compressive signs and symptoms, such as pain and edema in the left lower limb, and pelvic pain, among others.^3^^,^^8^^,^^29^^-^34

Studies show that the prevalence of this compressive phenomenon varies from 22 to 32% and that it is more common among females in the age range from 20 to 44 years.^3^^,^^8^^,^^27^^,^^29^^,^^31^^-^34 In our study, this compressive phenomenon was only detected in 15.8% of the sample, but, in agreement with the literature, LCIV compression was significantly more frequent in women than men and the age of those with compression was significantly lower than those without, at a mean of 45.9 years.

**Figure 4:**
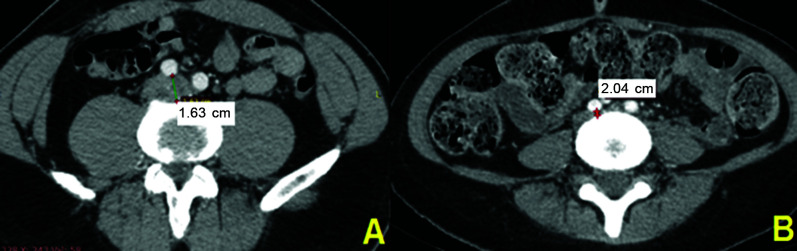
Computed tomography with intravenous contrast demonstrating measurement of the diameter of the left common iliac vein between the right common iliac artery and the spinal column. (A) Diameter of the left common iliac vein in a patient without venous compression (patient n 10); (B) Diameter of the left common iliac vein in a patient with venous compression (patient n 24).

Other studies describe that the mean diameter of the LCIV in patients without compression varies in the range of 7.5 mm to 13.1 mm, while the mean diameter in patients with DVT associated with May-Thurner Syndrome varies from 2.5 mm to 3.7 mm.^30^^,^^32^^,^^35^^,^^36^ It is also stated that a LCIV diameter < 4 mm is equivalent to approximately 70% of compression of the venous lumen, with a strong relationship with DVT and other symptoms of the syndrome.^31^^,^^33^^,^^35^^,^^36^ In our study, the mean LCIV diameter in patients without compression was 7.9 mm and in patients with compression mean diameter was 2.6 mm, which is in agreement with the studies mentioned. Figure 4 shows a comparison between patients with and without compression of the LCIV. In order to define the incidence and prevalence of these syndromes, obtaining greater diagnostic precision and helping with treatment decisions, it is necessary that future studies analyze correlations between radiological findings and the clinical status of patients. The lack of such as analysis is a limitation related to the retrospective nature of the current study.

## CONCLUSIONS

The prevalence of the nutcracker phenomenon was 24.2% and prevalence of LCIV compression was 15.8%, according to the radiological criteria adopted in this study. The rate of occurrence of LRV compression was not statistically different between men and women, but was most prevalent among individuals with a mean age of 48.8 years, while compression of the LCIV was more frequent among women aged approximately 45.9 years.

The mean diameter of the LCIV vein among patients with compression was 2.67 mm and mean diameter was 7.9 mm among patients without compression. Among patients with radiological criteria for LRV compression, the mean aortomesenteric angle was 32.8° and the mean hilar/aortomesenteric diameter ratio was 2.5. In patients without criteria, the mean aortomesenteric angle was 72.7° and the mean hilar/aortomesenteric diameter ratio was 1.2. The aortomesenteric angle was the more frequently detected of these two criteria for compression.
