# Supplementary material for: Compression of left renal vein and left common iliac vein on CT scans: how often are they detected?
Source: J Vasc Bras. 2020 Jun 1;19:e20190121. doi: 10.1590/1677-5449.190121 (PMC8202167; doi:10.1590/1677-5449.190121)
Supplement: Translation

# Translation: Compressões das veias renal e ilíaca comum esquerdas em tomografias computadorizadas: com que frequência são detectadas?

**Authors:** Adenauer Marinho de Oliveira Góes, Rafaela de Souza Araújo, Ismari Perini Furlaneto, Waldonio de Brito Vieira

**Affiliations:** 1 Centro Universitário do Estado do Pará – CESUPA, Habilidades Cirúrgicas e Cirurgia Vascular, Belém, PA, Brasil.; 2 Centro Universitário do Estado do Pará – CESUPA, Curso de Medicina, Belém, PA, Brasil.; 3 Clínica DIA/Hospital Amazônia, Radiologia, Belém, PA, Brasil.

**Keywords:** síndrome do quebra-nozes, síndrome de May-Thurner, tomografia computadorizada, veia ilíaca, compressão

## Abstract

**Contexto:**

As síndromes de *nutcracker* e May-Thurner são raras e, apesar de muitas vezes subdiagnosticadas, podem causar sintomas limitantes de gravidade variável. Frequentemente são consideradas diagnóstico de exclusão e não há consenso na literatura quanto a prevalência, incidência e critérios diagnósticos.

**Objetivos:**

Estimar a frequência da compressão das veias ilíaca comum e renal esquerdas em tomografias computadorizadas de abdome e pelve.

**Métodos:**

Estudo descritivo, quantitativo e transversal. Para veia renal esquerda, foram considerados como critérios de compressão a relação diâmetro hilar/aortomesentérico > 4 e o ângulo aortomesentérico < 39° e, para veia ilíaca comum esquerda, o diâmetro < 4 mm.

**Resultados:**

Foram analisadas tomografias computadorizadas de 95 pacientes; destes, 61% eram mulheres e 39% eram homens. A compressão da veia renal esquerda foi encontrada em 24,2% da amostra, com idade média de 48,8 anos, ocorrendo em 27,6% das mulheres e 18,9% dos homens (p = 0,3366). A compressão da veia ilíaca comum esquerda foi detectada em 15,7% da amostra, com idade média de 45,9 anos, ocorrendo em 24,10% das mulheres e 2,7% dos homens (p = 0,0024). Em 7,4% dos pacientes, ambas compressões venosas foram detectadas.

**Conclusões:**

A compressão da veia renal esquerda ocorreu em mulheres e homens com frequência semelhante, enquanto a compressão da veia ilíaca comum esquerda foi mais frequente em mulheres. Ambas as compressões venosas foram mais frequentemente encontradas em pacientes com idade entre 41 e 50 anos.

## INTRODUÇÃO

A síndrome do *nutcracker* (quebra-nozes, em português) é considerada rara. Ela corresponde ao conjunto de sinais e sintomas ocasionados pela compressão da veia renal esquerda (VRE) por uma redução do ângulo entre a aorta abdominal e a artéria mesentérica superior^1^^,^^2^. Uma variante menos frequente ocorre na presença de uma VRE retroaórtica, comprimida entre a aorta e o corpo vertebral (síndrome do *nutcracker* posterior)^3^^,^^4^. A síndrome foi descrita primeiramente por Schepper^5^ em 1972 e tem como achados clínicos mais comuns hematúria, dor pélvica, varizes pélvicas e proteinúria ortostática^6^^,^^7^. Pode evoluir com dor pélvica crônica, infertilidade e insuficiência renal^8^^,^^9^. A compressão venosa radiologicamente detectada, porém não associada a sintomas, é denominada fenômeno do *nutcracker*.

Para o diagnóstico radiológico, a tomografia computadorizada (TC) com contraste endovenoso é frequentemente utilizada por ser um exame não invasivo, relativamente barato e amplamente disponível. Na TC, podem ser medidos o ângulo aortomesentérico no plano sagital e a relação entre os diâmetros hilar e aortomesentérico da VRE no plano axial^10^^,^^11^. Um ângulo aortomesentérico < 39°^12^^,^^13^ e uma medida da relação diâmetro hilar/aortomesentérico > 4 são considerados critérios diagnósticos para a compressão da VRE^6^^,^^14^.

Outra situação anatômica que promove uma compressão venosa é a compressão da veia ilíaca comum esquerda (VICE) pela artéria ilíaca comum direita contra o promontório, que foi primeiramente descrita por Virchow^15^ em 1857 e posteriormente caracterizada como uma “síndrome”, detalhada em um estudo publicado por May e Thurner^16^ em 1958. Os principais achados clínicos são trombose venosa profunda iliofemoral, dor, varizes, edema, eczema venoso e úlceras de estase venosa no membro inferior esquerdo^8^^-^10. A TC tem alta sensibilidade e especificidade para o diagnóstico dessa síndrome^8^.

A síndrome de May-Thurner é classicamente descrita como mais prevalente em mulheres entre a terceira e quarta décadas de vida e pode estar associada a até 49% dos casos de trombose venosa profunda (TVP) no membro inferior esquerdo^4^^,^^15^^,^^17^. Nos estudos pioneiros de May e Thurner^16^, foi encontrada uma prevalência desta compressão venosa em 22% dos 430 cadáveres estudados.

Já a prevalência da síndrome do *nutcracker* permanece discutível devido à heterogeneidade de critérios diagnósticos e grande variedade de sintomas. Alguns estudos demonstram igual prevalência entre os sexos, ocorrendo principalmente em jovens e em pessoas com baixo índice de massa corporal^10^^,^^12^.

Os objetivos da pesquisa foram estimar a frequência da compressão das veias renal e ilíaca comum esquerdas em pacientes que realizaram TC de abdome e pelve; avaliar se a detecção da compressão destas veias é mais frequente em determinado sexo ou faixa etária; avaliar os diâmetros das respectivas veias em pacientes portadores e não portadores dos critérios de compressão e determinar qual dos critérios radiológicos para a compressão da VRE é mais frequentemente encontrado.

## MÉTODOS

Foi realizado um estudo descritivo quantitativo do tipo transversal, avaliando a prevalência da compressão da VRE (fenômeno do *nutcracker*) e da VICE em TC de abdome e pelve realizadas entre janeiro de 2017 e janeiro de 2018. A amostra foi de conveniência, obtida a partir de todos os exames disponibilizados por um serviço de radiologia parceiro da instituição de ensino correspondentes ao período do estudo após aplicação dos critérios de inclusão e exclusão. As tomografias computadorizadas com contraste endovenoso foram realizadas em tomógrafo GE Healthcare de 16 canais, com matriz de resolução de 512 × 512 e espessura de corte de 1,25 mm.

Os critérios de inclusão compreenderam as tomografias com contraste endovenoso de pacientes de ambos os sexos e idade mínima de 18 anos. Já os critérios de exclusão incluíram achados tomográficos suspeitos de malignidade que pudessem contribuir para a compressão venosa, malformações venosas renais e pélvicas e presença de stents em VICE e VRE.

Com auxílio do *software* RadiAnt DICOM viewer 4.6.9, foi calculada a relação entre o diâmetro da VRE ao nível hilar e ao nível do ângulo aortomesentérico em cortes axiais e foi medido o ângulo aortomesentérico nos cortes sagitais. Para a VICE, foi realizada medida no ponto de menor diâmetro entre a artéria ilíaca comum direita e o corpo vertebral adjacente.

Os critérios adotados para compressão da VRE foram os seguintes: relação diâmetro hilar/aortomesentérico > 4 e ângulo aortomesentérico < 39°. Para compressão da VICE, foi considerado como critério o diâmetro < 4 mm.

A normalidade das distribuições foi verificada pelo teste D’Agostino-Pearson, sendo utilizados o teste t de Student para amostras independentes (distribuições paramétricas), o teste de Mann-Whitney, o teste do qui-quadrado de aderência ou o teste G para amostras independentes (distribuições não paramétricas). Todos os testes foram executados com o auxílio do programa BioEstat 5.4, e considerou-se o valor de p ≤ 0,05 para significância estatística. A pesquisa foi aprovada pelo comitê institucional de ética em pesquisa sob o protocolo 2.683.725.

## RESULTADOS

Foram analisadas 95 tomografias computadorizadas de abdome e pelve, sendo que dois pacientes foram excluídos da análise do ângulo aortomesentérico por apresentarem VRE retroaórtica. A média de idade dos pacientes foi 53,70 anos ± 14,90 anos, variando entre 21 e 83 anos; em relação à faixa etária, a maioria dos pacientes possuía 61 anos ou mais (p = 0,0002; Tabela 1). Embora as pacientes do sexo feminino tenham predominado na amostra (p = 0,0312; Tabela 1), não houve diferença estatisticamente significativa entre a proporção de homens e mulheres dentro de cada faixa etária estudada (p = 0,5295; dados não mostrados).

**Tabela 1:** Distribuição dos pacientes segundo o sexo e a faixa etária.

**Variável**	**n**	**%**	**p-valor** ^*^
**Sexo**			
Masculino	37	39,00	0,0312†
Feminino	58	61,00	
**Faixa etária (anos)**			
21-30	08	8,40	0,0002^†^
31-40	11	11,60	
41-50	18	18,90	
51-60	25	26,30	
≥ 61	33	34,80	

* Qui-quadrado de aderência;

† Estatisticamente significativo;

n: número de pacientes.

A Tabela 2 apresenta os diâmetros médios da VRE na porção hilar e ao nível do ângulo aortomesentérico, os valores médios da relação diâmetro hilar/aortomesentérico, as medidas do ângulo aortomesentérico e os diâmetros da VICE no ponto de maior compressão. No que se refere à compressão da VRE, observou-se ângulo aortomesentérico < 39° em 22 de 93 pacientes (23,70%) e relação diâmetro hilar/aortomesentérico > 4 em 2 de 95 (2,10%) pacientes. Um de 95 (1,10%) pacientes possuía os dois critérios positivos, totalizando 23 de 95 (24,2%) pacientes com um ou mais critérios que definem o fenômeno do *nutcracker.* A compressão da VICE (diâmetro < 4 mm) foi identificada em 15 de 95 (15,80%) pacientes. Em 7 de 95 (7,4%) pacientes, foram detectados um ou mais critérios tomográficos para compressão da VRE e da VICE simultaneamente.

**Tabela 2:** Diâmetros das veias renal esquerda e ilíaca comum esquerda, relação entre os diâmetros da veia renal esquerda no segmento hilar e na topografia do ângulo aortomesentérico, medidas do ângulo aortomesentérico.

**Variável**	**Média ± desvio padrão**	**Mínimo-máximo**
Diâmetro hilar da VRE (mm)	8,37±1,94	3,25-13,40
Diâmetro aortomesentérico da VRE (mm)	6,63±2,58	1,18-16,10
Relação diâmetro hilar/aortomesentérico	1,53±0,93	0,51-6,66
Ângulo aortomesentérico (graus)	61,12±24,53	17,60-124,70
Diâmetro VICE (mm)	7,74±3,89	1,31-22,80

mm = milímetros; VRE = veia renal esquerda; VICE = veia ilíaca comum esquerda.

A Tabela 3 apresenta a comparação da relação diâmetro hilar/aortomesentérico e do ângulo aortomesentérico em pacientes com e sem compressão da VRE. Nota-se relação diâmetro hilar/aortomesentérico significativamente menor entre os pacientes com compressão da VRE (p < 0,0001) e ângulo aortomesentérico significativamente maior entre os pacientes sem essa compressão (p < 0,0001).

**Tabela 3:** Comparação da relação diâmetro hilar/aortomesentérico e do ângulo aortomesentérico em pacientes com e sem compressão da veia renal esquerda.

**Variável**	**Compressão da veia renal esquerda**		**p-valor**
	**Presente**	**Ausente**	
**Relação diâmetro hilar/aortomesentérico**	**n = 23**	**n = 72**	
Média ± desvio padrão	2,53±1,29	1,21±0,42	< 0,0001†
Mínimo-máximo	0,69-6,66	0,51-2,51	
IC95%	1,97-3,09	1,11-1,30	
**Ângulo aortomesentérico (graus)**	**n = 23**	**n = 70** a	< 0,0001^†^
Média ± desvio padrão	32,71±15,43	70,45±19,21	
Mínimo-máximo	17,60-97,70	41,00-83,70	
IC95%	26,03-39,38	65,87-75,03	

Teste de Mann-Whitney.

† Estatisticamente significativo;

n: número de pacientes;

a n = 2 pacientes excluídos dessa comparação por possuírem VRE (veia renal esquerda) retroaórtica; IC95% = intervalo de confiança de 95%.

No que diz respeito à presença da compressão da VRE relacionada ao gênero, não foi observada diferença estatisticamente significante (p = 0,3666; Tabela 4). A média de idade entre os portadores e não portadores da compressão, no entanto, foi semelhante (p = 0,0666; Tabela 5).

**Tabela 4:** Distribuição dos pacientes de acordo com o sexo e a presença das compressões venosas investigadas.

**Variável**	**Sexo**		**p-valor**
	**Feminino n; %**	**Masculino n; %**	
**Compressão da VRE**			
Presença	16; 27,60	07;18,90	0,3366
Ausência	42; 72,40	30; 81,10	
**Compressão da VICE**			
Presença	14; 24,10	01; 2,70	0,0024†
Ausência	44; 75,90	36; 97,30	
**Ambas as compressões**			
Presença	06; 10,30	01; 2,70	-

Teste G de independência. n: número de pacientes.

† Estatisticamente significativo; VRE = veia renal esquerda; VICE = veia ilíaca comum esquerda.

**Tabela 5:** Valores médio, mínimo e máximo e desvio padrão da idade de acordo com a presença das compressões investigadas.

**Variável**	**Idade (anos)**	**Mínimo-máximo**	**p-valor**
	**Média ± desvio padrão**		
**Compressão da VRE**			
Presença	48,80±17,90	21-83	0,0666
Ausência	55,30±13,50	24-82	
**Compressão da VICE**			
Presença	45,90±15,20	24-77	0,0248†
Ausência	55,20±14,40	21-83	
**Ambas as compressões**			
Presença	51,60±16,50	26-77	0,6924
Ausência	53,90±14,80	21-83	

Teste t de Student.

† Estatisticamente significativo; VRE = veia renal esquerda; VICE = veia ilíaca comum esquerda.

Na Tabela 6, é possível verificar que o diâmetro da VICE é significativamente menor em pacientes classificados como portadores da compressão dessa veia (p < 0,0001). A compressão da VICE foi detectada com frequência significativamente maior em mulheres (p = 0,0024), e a média de idade dos pacientes que apresentaram esse fenômeno foi significativamente menor quando comparada com a idade média dos que não o manifestaram (p = 0,0248; Tabelas 4 e 5).

**Tabela 6:** Comparação do diâmetro da veia ilíaca comum esquerda em pacientes com e sem compressão da veia ilíaca comum esquerda.

**Variável**	**Compressão da veia ilíaca comum esquerda**		**p-valor** *
	**Presença**	**Ausência**	
**Diâmetro da veia ilíaca comum esquerda (mm)**	n = 15	n = 80	
Média ± desvio padrão	2,69±0,76	8,69±3,48	< 0.0001†
Mínimo-máximo	1,31-3,90	4,43-22,80	
IC95%	2,26-3,10	7,92-9,46	

* Teste de Mann-Whitney;

n: número de pacientes;

† Estatisticamente significativo; IC95% = intervalo de confiança de 95%; mm = milímetros.

## DISCUSSÃO

Neste estudo, embora a identificação radiológica de tais fenômenos compressivos tenha sido pesquisada, não foi possível estabelecer correlações clínicas, já que apenas o acesso às imagens de tomografia estava disponível Entre as 95 tomografias analisadas, 58 eram de mulheres (61%) e 37 eram de homens (39%). Essa proporção entre os sexos é semelhante à encontrada em diversos estudos envolvendo tomografias computadorizadas sobre o mesmo assunto, como o de Zhong et al.^18^, cuja amostra foi composta 75% por mulheres e 25% por homens, e de Narayan et al.^19^, com composição amostral de 59% de mulheres e 41% de homens. Segundo estudo de Levorato et al.^20^, esse padrão pode dever-se à maior procura dos serviços de saúde por pacientes do gênero feminino.

Com respeito à síndrome do *nutcracker*, durante a revisão de literatura observou-se grande divergência quanto aos pontos de corte adotados para o ângulo aortomesentérico, havendo estudos que consideraram desde 25 até 45°^4^^,^^7^^,^^8^^,^^12^^,^^13^^,^^21^^,^^22^ e relação diâmetro hilar/aortomesentérico variando entre > 4 e > 4,9^6^^,^^7^^,^^12^^-^14^,^^21^. Nesta pesquisa, foram considerados para a presença da compressão da VRE os critérios de relação hilar/aortomesentérica > 4 e ângulo aortomesentérico < 39°.

Alguns estudos descrevem que a compressão da VRE é mais frequente em mulheres jovens entre a segunda e quarta décadas de vida^3^^,^^4^^,^^8^^,^^9^^,^^23^. Porém, outros estudos sugerem não haver diferença estatisticamente significativa na distribuição entre os sexos, tanto na síndrome quanto no fenômeno do *nutcracker* 11^,^^12^^,^^18^^,^^21^^,^^22^^,^^24^^,^^25^. Nosso estudo não demonstrou diferença estatisticamente significativa de ocorrência entre os sexos. Yun et al.^21^ demonstrou que a compressão venosa estava presente em 37,5% dos pacientes; já no estudo de Poyraz et al.^11^, a prevalência descrita foi de 10,4%. Essa divergência pode ser devida à falta de uniformidade nos pontos de corte entre os autores. Em nosso estudo, foi encontrada uma frequência de 24,2%.

No estudo de Yun et al.^21^, a média de idade encontrada para pacientes com compressão da VRE foi de 36,8 ± 14,3 anos e, no de Kim et al.^13^, a média foi de 23,9 ± 4,6 anos. A média de idade que encontramos para pacientes portadores de compressão da VRE foi de 48,8 anos.

O ângulo aortomesentérico médio encontrado no estudo de Yun et al.^21^ foi de 20° em pacientes com a síndrome do *nutcracker* e de 25° em pacientes assintomáticos, enquanto Zhong et al.^18^ detectou ângulo de 32,3° ± 7,6° em pacientes com a síndrome do *nutcracker*. Em nosso estudo, o ângulo aortomesentérico médio nos portadores de compressão foi 27,3°, aproximando-se dos resultados visualizados na literatura.

Encontramos relação do diâmetro hilar/aortomesentérico > 4 em apenas 2 pacientes; já o ângulo aortomesentérico < 39° foi detectado em 22 dos 93 pacientes submetidos a esta análise, sugerindo uma maior sensibilidade desse critério. Como o critério da relação diâmetro hilar/aortomesentérico é encontrado com frequência menor, este pode ser mais específico e, portanto, mais valorizado quando detectado em pacientes com clínica compatível com a síndrome. Essa grande diferença da frequência entre os dois critérios pode ter ocorrido por termos padronizado um ângulo aortomesentérico < 39° para compressão da VRE. Caso houvéssemos adotado ângulos menores, como outros autores, essa disparidade poderia ter sido menor; no entanto, o valor a ser adotado diverge amplamente na literatura^4^^,^^7^^,^^8^^,^^12^^,^^13^^,^^21^^,^^22^ e é um dos pontos ainda sem consenso acerca deste tema.

Zhong et al.^18^ obtiveram uma média de relação do diâmetro hilar/aortomesentérico de 3,4; já Kim et al.^13^ descrevem que a média do diâmetro da relação hilar/aortomesentérica em sua pesquisa foi de 5 ± 1,7, ambos os estudos em pacientes sintomáticos para a síndrome do *nutcracker*. Em nosso estudo, a média da relação diâmetro hilar/aortomesentérico nos pacientes considerados portadores de compressão (por apresentarem ângulo aortomesentérico < 39°) foi de 2,1.

Essa discordância pode ser atribuída ao fato de que os pacientes dos estudos supracitados possuíam o diagnóstico da síndrome do *nutcracker*, enquanto os pacientes do nosso estudo foram avaliados apenas por achados radiológicos incidentais de compressão. Não foram encontrados estudos que comparassem a relação diâmetro hilar/aortomesentérico em indivíduos com e sem sintomas.

Detectamos em um dos pacientes a compressão da VRE, porém sem estreitamento do ângulo aortomesentérico, como é classicamente relatado (paciente cuja tomografia é apresentada na Figura 1). Esse achado pode ocorrer pela interposição duodenal, descrita como uma causa de compressão da VRE com um ângulo aortomesentérico normal^14^.

**Figura 1:** Tomografia computadorizada com contraste endovenoso (paciente n 67). (A) Diâmetro da veia renal esquerda no hilo renal; (B) Diâmetro da veia renal esquerda na topografia do ângulo aortomesentérico; (C) Aferição do ângulo aortomesentérico em corte sagital.

Foi detectada, em dois pacientes, uma variação anatômica da VRE retroaórtica, como mostra a Figura 2. Essa variação permite a avaliação da compressão apenas estabelecendo-se a relação entre o diâmetro hilar e o diâmetro da veia no ponto máximo de compressão entre a aorta e a vértebra adjacente, visto que a VRE desses pacientes não tem seu trajeto entre o ângulo aortomesentérico^6^. Esses pacientes foram excluídos dos cálculos envolvendo o ângulo aortomesentérico, porém, devido ao mecanismo hemodinâmico semelhante, foram avaliados segundo o mesmo ponto de corte aplicado para avaliação da relação diâmetro hilar/aortomesentérico nos demais pacientes.

**Figura 2:** Tomografia computadorizada com contraste endovenoso (paciente n 54). (A) Diâmetro da veia renal esquerda no hilo renal; (B) Diâmetro da veia renal esquerda na topografia retroaórtica.

Encontramos o valor para a relação do diâmetro hilar/aortomesentérico > 4 em apenas dois pacientes. Apenas um paciente possuía os dois critérios positivos, como demonstrado na Figura 3.

**Figura 3:** Tomografia computadorizada com contraste endovenoso (paciente n 71). (A) Diâmetro da veia renal esquerda no hilo renal; (B) Diâmetro da veia renal esquerda na topografia do ângulo aortomesentérico; (C) Aferição do ângulo aortomesentérico em corte sagital.

A síndrome de May-Thurner se refere à compressão da VICE entre a artéria ilíaca comum direita e a vértebra lombar adjacente^26^^-^28, provocando sinais e sintomas compressivos, como dor e edema em membro inferior esquerdo, dor pélvica, entre outros^3^^,^^8^^,^^29^^-^34.

Estudos mostram que a prevalência desse fenômeno compressivo varia entre 22 e 32% e é mais frequente em indivíduos do sexo feminino na faixa etária entre 20 e 44 anos^3^^,^^8^^,^^27^^,^^29^^,^^31^^-^34. Em nossa pesquisa, esse fenômeno compressivo foi detectado em apenas 15,8% da amostra; porém, em consonância com a literatura, a compressão da VICE foi significativamente mais frequente em mulheres do que em homens, e a idade dos que possuem a compressão foi significativamente menor em relação aos que não possuem, sendo, em média, de 45,9 anos.

Outras pesquisas descrevem que a média do diâmetro da VICE em pacientes sem compressão varia entre 7,5 mm e 13,1 mm, e a média em pacientes com TVP associada à síndrome de May-Thurner varia de 2,5 mm a 3,7 mm^30^^,^^32^^,^^35^^,^^36^. É descrito ainda que um diâmetro da VICE < 4 mm equivale a aproximadamente 70% de compressão do lúmen venoso, relacionando-se fortemente com a ocorrência de TVP e outros sintomas da síndrome^31^^,^^33^^,^^35^^,^^36^. Em nosso estudo, a média do diâmetro da VICE em pacientes sem compressão foi de 7,9 mm e dos pacientes com compressão foi de 2,6 mm, estando de acordo com os estudos mencionados. A Figura 4 mostra a comparação entre pacientes com e sem compressão da VICE. Para definição da incidência e prevalência das síndromes, obtendo maior precisão no diagnóstico e auxiliando na decisão terapêutica, será necessário que, em futuras pesquisas, se realize a correlação entre os achados radiológicos e o quadro clínico dos pacientes, uma limitação associada ao caráter retrospectivo da pesquisa atual.

**Figura 4:** Tomografia computadorizada com contraste endovenoso demonstrando aferição do diâmetro da veia ilíaca comum esquerda entre a artéria ilíaca comum direita e a coluna vertebral. (A) Diâmetro da veia ilíaca comum esquerda em paciente sem compressão venosa (paciente n 10); (B) Diâmetro da veia ilíaca comum esquerda em paciente com compressão venosa (paciente n 24).

## CONCLUSÕES

A prevalência do fenômeno do *nutcracker* foi de 24,2% e da compressão da VICE de 15,8% pelos critérios radiológicos adotados neste estudo. A ocorrência da compressão da VRE não foi estatisticamente diferente entre homens e mulheres e foi mais prevalente em indivíduos com idade média de 48,8 anos, enquanto a compressão da VICE foi mais frequente em mulheres de aproximadamente 45,9 anos.

O diâmetro médio da veia VICE, em portadores de compressão, foi de 2,67 mm e de 7,9 mm nos pacientes sem compressão. Em pacientes com critérios radiológicos de compressão da VRE, os valores médios foram 32,8° para o ângulo aortomesentérico e 2,5 para relação diâmetro hilar/aortomesentérico. Já em pacientes sem os critérios, os valores médios foram de 72,7° para o ângulo aortomesentérico e 1,2 para a relação diâmetro hilar/aortomesentérico. Entre estes critérios de compressão, o relacionado à medida do ângulo aortomesentérico foi o mais frequentemente detectado.
